# Network Pharmacology, Molecular Docking, and Molecular Dynamics Simulations to Elucidate the Potential Mechanism of Ermiao San in Osteoarthritis

**DOI:** 10.1002/fsn3.71287

**Published:** 2025-12-10

**Authors:** Zhenyu Song, Jincheng Huang, He Zhu, Xu Li, Qianqian Cao, Liyang Yang, Meng Zhang, Hongkai Wang, Haoyue Sun

**Affiliations:** ^1^ The Second Affiliated Hospital of Guilin Medical University Guilin Guangxi China; ^2^ Henan Provincial People's Hospital Henan University People's Hospital, Zhengzhou University People's Hospital Zhengzhou Henan China; ^3^ North Henan Medical University Xinxiang Henan China; ^4^ Fuwai Central China Cardiovascular Hospital, Henan Cardiovascular Hospital and Zhengzhou University, Henan Province People's Hospital Zhengzhou Henan China

**Keywords:** EMS, molecular docking, molecular dynamics simulations, network pharmacology, osteoarthritis

## Abstract

This study aims to identify the active components and molecular mechanisms of Ermiao San (EMS) in the treatment of osteoarthritis (OA) through network pharmacology, molecular docking, and molecular dynamics simulations. EMS compounds and their targets were retrieved from TCMSP; OA‐related targets were collected from five public databases. Potential drug‐disease target interactions were analyzed using STRING 12.0 and Cytoscape 3.10.2. Functional and pathway enrichment analyses were performed on the 90 overlapping targets. Molecular docking was performed with CB‐Dock2 and LigPlot+ v2.2.8 platforms, followed by a comprehensive evaluation of key compounds via SwissADME. We identified 46 active chemicals, 187 EMS‐specific targets, and 1718 OA‐related targets, with 90 overlapping targets. Molecular docking and molecular dynamics simulations analysis revealed a strong binding potential of key EMS compounds to target proteins. These findings suggest that EMS exerts its anti‐OA effects through multicomponent, multi‐target, and multipathway interactions.

## Graphical abstract

Integrated network pharmacology, molecular docking, and dynamics simulations revealed the anti‐osteoarthritis mechanism of Ermiao San. Key bioactive compounds (e.g., quercetin) synergistically target core proteins (e.g., TNF and IL‐6), modulating critical pathways (e.g., TNF signaling) to inhibit inflammation and cartilage degradation, demonstrating a multicomponent, multi‐target therapeutic action.

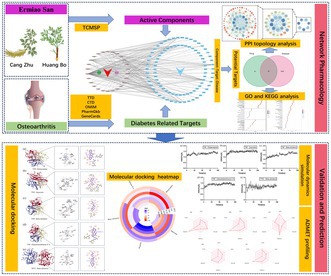

## 1 Introduction

Osteoarthritis (OA) is a degenerative joint disease primarily caused by aging, obesity, overuse, trauma, congenital joint abnormalities, and joint deformities (Jiang 2022). Clinically, it is characterized by joint pain, stiffness, deformity, and functional impairment. Current data show that approximately 250 million people worldwide suffer from OA, with far‐reaching consequences for patients, healthcare systems, and socioeconomics (Glyn‐Jones et al. 2015). Conventional drug therapy for OA usually only provides symptomatic relief, while effective methods for promoting cartilage regeneration and reversing the course of OA remain challenging. As a result, increasing attention has been given to herbal therapies, which have demonstrated significant efficacy in treating OA (Xiang et al. 2022).

Network pharmacology offers a robust framework for understanding the systemic effects of drugs by analyzing interactions between multiple biological networks. For example, Demir et al. (2025) demonstrated esculetin's cardioprotective role against doxorubicin‐induced toxicity via multi‐target actions on enzymes like aldose reductase and acetylcholinesterase, using gene expression analysis and molecular docking. Similarly, Tokalı, Demir, Tokalı, et al. (2025) developed quinazolinone‐thiazolidinedione hybrids as dual inhibitors of α‐glycosidase and aldose reductase, employing synthetic, in vitro, and in silico approaches to validate their efficacy. Unlike traditional drug discovery methods that focus on single targets, network pharmacology considers the broader biological context, enabling the identification of multi‐target therapies such as Ermiao San (EMS), a traditional Chinese medicine formula (Nogales et al. 2022). It is increasingly used in drug development, based on the concept that complex diseases like OA result not from single‐gene mutations but from disruptions in biological network systems caused by multiple genetic alterations (Zhang et al. 2022). Molecular docking is a computational simulation technique used to model interactions between molecules and proteins at the atomic level (Liu et al. 2022). This method predicts the conformations of ligands and receptors and calculates parameters such as affinity to evaluate binding interactions. Tokalı, Demir, Tokalı, et al. (2025) applied docking and dynamics simulations to elucidate the binding mechanisms of thiazolidine‐2,4‐dione derivatives as dual α‐glucosidase and aldose reductase inhibitors, emphasizing hydrogen bonding and hydrophobic interactions. Similarly, docking analysis was used to guide the structural optimization of quinazolin‐4 (3H)‐one derivatives with improved binding affinity toward target enzymes (Tokalı, Demir, Ateşoğlu, et al. 2025). Its accuracy and cost‐effectiveness make it valuable for drug design and elucidating biochemical pathways.

Traditional Chinese medicine (TCM) has a long history in treating OA. EMS, developed by Zhu Danxi during the Song Dynasty and recorded in “Danxi's Heart Method,” is a TCM formula made from Rhizoma Atractylodis (Cang Zhu) and Cortex Phellodendri (Huang Bo) in a 1:1 ratio (Guo et al. 2022). This formula is known for its ability to clear heat, dry dampness, expel damp‐heat, and improve joint blood circulation and qi stagnation (Geng et al. 2022). EMS is primarily used to treat conditions such as lower limb weakness, redness, swelling, heat pain in the feet and knees, and damp‐heat related symptoms, including lower extremity sores, yellow and scanty urine, and greasy yellow tongue coating (Liu et al. 2024).

Pharmacological studies have demonstrated the multifaceted effects of the TCM formula EMS, including its anti‐inflammatory, antioxidant, and anti‐apoptotic activities (Guo et al. 2022; Geng et al. 2022; Liu et al. 2024). While previous research and recent network pharmacology analyses have explored EMS for other conditions, a comprehensive, validated analysis of its mechanism specifically for OA is lacking. The novelty of this study is its integrated use of network pharmacology and molecular docking to systematically elucidate the “multicomponent, multi‐target, multipathway” therapeutic mechanism of EMS against OA.

## 2 Materials and Methods

### 2.1 Identification of Active Compounds and Associated Targets

Active ingredients of Cangzhu and Huangbo were sourced from the Traditional Chinese Medicine Systems Pharmacology Database and Analysis Platform (TCMSP) (https://old.tcmsp‐e.com/tcmsp.php). To identify compounds with potential for oral bioavailability and drug‐like properties, screening criteria based on established network‐pharmacology protocols were applied to select ingredients exhibiting drug‐likeness ≥ 0.18 and oral bioavailability ≥ 30%, and the resulting data were used for subsequent analyses (Zhang et al. 2019). These data were subsequently used for further analysis. The targets corresponding to Cangzhu and Huangbo were also retrieved from the TCMSP database, a specialized online platform that offers essential data on Chinese herbal medicines, such as herbal components, targets, and ingredient‐target interaction networks. To enable further analysis, target proteins from the TCMSP database were converted into gene IDs through the UniProt database. The gene targets obtained were subsequently deduplicated and prepared for further processing.

### 2.2 Predicting Targets of OA

Searches were conducted using GeneCards (https://www.genecards.org/), CTD (https://sctdbase.org/), PharmGKB (https://www.pharmgkb.org/), TTD (https://db.idrblab.net/ttd/), and OMIM (https://www.omim.org/) with the keyword “OA” to identify disease‐related targets in *Homo sapiens* . Targets retrieved from GeneCards with a relevance score exceeding 1 were chosen as potential candidates. For the OMIM, CTD, TTD, and PharmGKB datasets, all targets associated with OA were considered for inclusion.

### 2.3 Network Construction

#### 2.3.1 Drug‐Compound‐Target‐Disease Network

The common targets and active compounds were loaded into Cytoscape 3.10.2 to build the Drug‐Compound‐Target‐Disease network. The “Network Analyzer” tool was then employed to assess the topological characteristics of the network.

#### 2.3.2 Protein–Protein Interaction (PPI) Network

Protein–protein interactions were examined using STRING version 12.0 (https://string‐db.org/). To investigate the mechanism of EMS in OA treatment, the intersecting targets were imported into STRING, with a minimum combined score set at 0.900. Interaction data specific to *Homo sapiens* were extracted, and targets without interactions were removed. The resulting data were stored as SIF files. Core targets were determined through cluster analysis using the cyto NCA plug‐in in Cytoscape.

### 2.4 GO and KEGG Pathway Enrichment Analyses

Gene Ontology (GO) enrichment analysis was performed using the “clusterProfiler” package in R (R Foundation for Statistical Computing). This analysis focused on three categories: Biological Process (BP), Cellular Component (CC), and Molecular Function (MF), with significant annotations selected based on a *p*‐value of less than 0.05. Additionally, Kyoto Encyclopedia of Genes and Genomes (KEGG) pathway enrichment analysis was conducted to identify key signaling pathways related to the anti‐OA effects of EMS. The enriched results were visualized using the “ggplot2” package in R.

### 2.5 In Silico Studies

#### 2.5.1 Molecular Docking

The 3D structures of the selected compounds were retrieved in .sdf format from the PubChem database (https://pubchem.ncbi.nlm.nih.gov/), enabling further molecular docking and analysis of binding affinities. The crystal structures of human IL‐6 (PDB ID: 1ALU), ESR1 (PDB ID: 1SJ0), IL1B (PDB ID: 1I1B), AKT1 (PDB ID: 2UVM), MAPK1 (PDB ID: 3D44), TNF (PDB ID: 2E7A) and TP53 (PDB ID: 1UOL) were obtained from the RCSB Protein Data Bank (https://www.rcsb.org). The molecular docking procedures were performed on the CB‐Dock2 platform (https://cadd.labshare.cn/cb‐dock2/index.php) (Liu et al. 2022). The system automatically carried out structural preprocessing, including hydrogenation, side‐chain completion, and removal of water molecules and heteroatoms for the protein, as well as charge assignment and three‐dimensional conformation generation for the ligand. Binding site prediction was conducted using a curvature‐based cavity detection algorithm under a blind docking workflow. The conformation with the lowest binding score computed by Auto Dock Vina version 1.1.2 was selected as the optimal binding pose. The binding affinity was evaluated based on the docking energy (in kcal/mol) provided by the platform. The protein–ligand interaction patterns were visualized using the built‐in 3D viewer of CB‐Dock2.

#### 2.5.2 Analysis of Protein–Ligand Complex

The protein‐ligand complexes were stored in .pdb file format and analyzed with LigPlot+ v2.2.8, a tool used to visually depict interactions between proteins and ligands. LigPlot+ creates a 2D interaction map that highlights the amino acids involved and specifies the types and quantities of noncovalent interactions between the protein and ligand.

#### 2.5.3 Molecular Dynamics Simulation

YASARA version 10.3.16 was used to perform molecular dynamics simulations. This preprocessing involved removing non‐essential atoms, adding hydrogen atoms, and assigning protonation states at physiological pH (7.4) to ensure structural correctness. This optimized complex was subsequently subjected to a molecular dynamics simulation using the AMBER force field. Water molecules and ions were added according to the software's default protocols. Following energy minimization and system equilibration, a production molecular dynamics simulation was carried out for 100 ns. Parameters such as the root‐mean‐square deviation (RMSD) were recorded throughout the simulation trajectory. Finally, the simulation data were visualized and analyzed using GraphPad Prism version 9.0. Line graphs were generated to provide an intuitive interpretation of the temporal evolution of the measured parameters, facilitating the assessment of system stability.

#### 2.5.4 Pharmacokinetics Prediction

The in silico predicted ADMET (Absorption, Distribution, Metabolism, Excretion, and Toxicity) profiles of the screened compounds were evaluated using the SwissADME online server (Daina et al. 2017). SwissADME utilizes machine learning algorithms and molecular descriptor‐based models to predict a wide range of pharmacokinetic and drug‐like properties. In this study, SwissADME was employed to comprehensively assess key parameters. All predictions were carried out using the default settings of the platform.

## 3 Results

### 3.1 Prediction of Active Compounds and Targets of EMS

A total of 46 active components of EMS were selected from the TCMSP database based on the criteria of oral bioavailability (OB) ≥ 30% and drug‐likeness (DL) ≥ 0.18. Specifically, nine active components were identified from Cang Zhu, and 37 were from Huang Bo (Table 1). The TCMSP database identified 486 unique potential targets related to the active components of the studied compounds. Specifically, 59 targets were associated with Cangzhu, and 426 targets were linked to Huangbo. After removing duplicates, 187 distinct drug targets were identified.

**TABLE 1:** OB and DL of 46 active compounds in EMS.

Herb	MOL ID	Compound	OB/%	DL
Cangzhu	MOL000085	Beta‐daucosterol_qt	36.91	0.75
	MOL000088	Beta‐sitosterol 3‐O‐glucoside_qt	36.91	0.75
	MOL000092	Daucosterin_qt	36.91	0.76
	MOL000094	Daucosterol_qt	36.91	0.76
	MOL000173	Wogonin	30.68	0.23
	MOL000179	2‐Hydroxyisoxypropyl‐3‐hydroxy‐7‐isopentene‐2,3‐dihydrobenzofuran‐5‐carboxylic	45.2	0.2
	MOL000184	Nsc63551	39.25	0.76
	MOL000186	Stigmasterol 3‐O‐beta‐D‐glucopyranoside_qt	43.83	0.76
	MOL000188	3β‐acetoxyatractylone	40.57	0.22
Huangbo	MOL000098	Quercetin	46.43	0.28
	MOL000358	Beta‐sitosterol	36.91	0.75
	MOL000449	Stigmasterol	43.83	0.76
	MOL000622	Magnograndiolide	63.71	0.19
	MOL000762	Palmidin A	35.36	0.65
	MOL000785	Palmatine	64.6	0.65
	MOL000787	Fumarine	59.26	0.83
	MOL000790	Isocorypalmine	35.77	0.59
	MOL001131	Phellamurin_qt	56.6	0.39
	MOL001454	Berberine	36.86	0.78
	MOL001455	(S)‐canadine	53.83	0.77
	MOL001458	Coptisine	30.67	0.86
	MOL001771	Poriferast‐5‐en‐3beta‐ol	36.91	0.75
	MOL002636	Kihadalactone A	34.21	0.82
	MOL002641	Phellavin_qt	35.86	0.44
	MOL002643	Delta 7‐stigmastenol	37.42	0.75
	MOL002644	Phellopterin	40.19	0.28
	MOL002651	Dehydrotanshinone II A	43.76	0.4
	MOL002652	Delta7‐Dehydrosophoramine	54.45	0.25
	MOL002656	Dihydroniloticin	36.43	0.81
	MOL002659	Kihadanin A	31.6	0.7
	MOL002660	Niloticin	41.41	0.82
	MOL002662	Rutaecarpine	40.3	0.6
	MOL002663	Skimmianin	40.14	0.2
	MOL002666	Chelerythrine	34.18	0.78
	MOL002668	Worenine	45.83	0.87
	MOL002670	Cavidine	35.64	0.81
	MOL002671	Candletoxin A	31.81	0.69
	MOL002672	Hericenone H	39	0.63
	MOL002673	Hispidone	36.18	0.83
	MOL002894	Berberrubine	35.74	0.73
	MOL005438	Campesterol	37.58	0.71
	MOL006392	Dihydroniloticin	36.43	0.82
	MOL006401	Melianone	40.53	0.78
	MOL006413	Phellochin	35.41	0.82
	MOL006422	Thalifendine	44.41	0.73
	MOL013352	Obacunone	43.29	0.77

Abbreviations: DL, drug‐likeness; OB, oral bioavailability.

### 3.2 Prediction of Targets and Common Targets of OA

**FIGURE 1:**
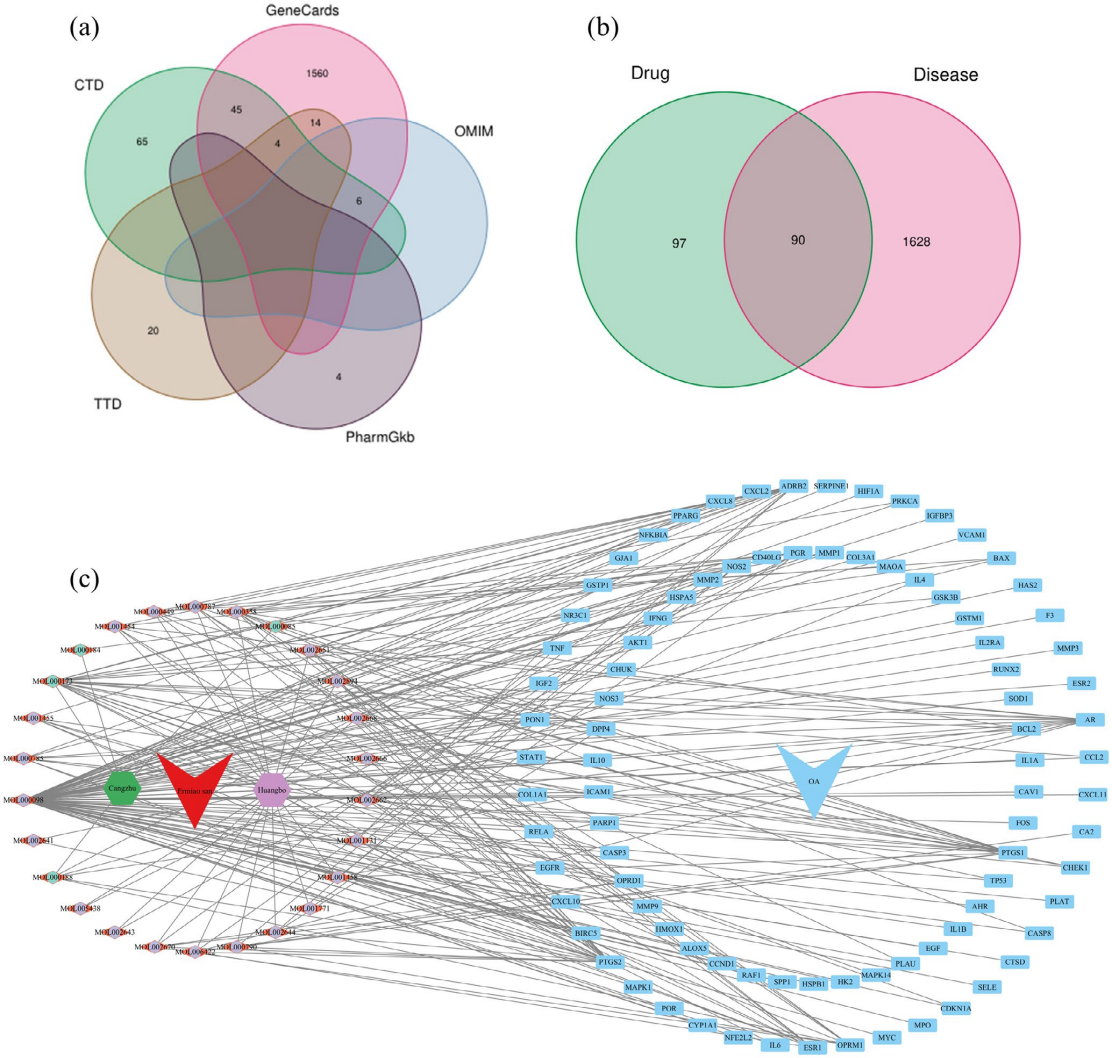
Identification of EMS and OA targets. (A) Venn diagram of targets from five OA‐related databases. (B) Venn diagram showing the overlap between EMS compound targets and OA‐related targets. (C) The “Herb‐Compound‐Target‐OA” network.

A total of 1718 OA‐related targets were retrieved from the GeneCards, OMIM, TTD, CTD, and PharmGKB databases after removing duplicates (Figure 1a). By intersecting these 1718 disease‐related targets with the 187 targets of the active compounds in EMS, 90 overlapping targets were identified (Figure 1b), which represent the primary potential targets of EMS for OA treatment.

### 3.3 Construction of the Interaction Network for Targets of EMS Active Compounds in OA

The data were imported into Cytoscape 3.10.2 to construct a visual “drug‐Compound‐Target‐OA” network of EMS, as shown in Figure 1c. The network consisted of 120 nodes and 234 edges. Cang Zhu and Huang Bo corresponded to 4 and 22 compounds and 31 and 73 targets, respectively. The top 5 targets with the highest degree values were Prostaglandin‐Endoperoxide Synthase 2 (PTGS2), Prostaglandin‐Endoperoxide Synthase 1 (PTGS1), Beta‐2 Adrenergic Receptor (ADRB2), Androgen Receptor (AR), and Estrogen Receptor Alpha (ESR1). The top five compounds with the highest degree values were quercetin, wogonin, beta‐sitosterol, dehydrotanshinone II A, and rutaecarpine, interacting with 78, 25, 12, 9, and 9 targets, respectively (Table 2).

**TABLE 2:** The top 5 active components in the EMS.

MOL	Active components	OB (%)	DL	Herb	Degree
MOL000098	Quercetin	46.43	0.28	Huangbo	78
MOL000173	Wogonin	30.68	0.23	Cangzhu	25
MOL000358	Beta‐sitosterol	36.91	0.75	Huangbo	12
MOL002651	Dehydrotanshinone II A	43.76	0.4	Huangbo	9
MOL002662	Rutaecarpine	40.3	0.6	Huangbo	9

### 3.4 PPI Network of EMS in the Treatment of OA

**FIGURE 2:**
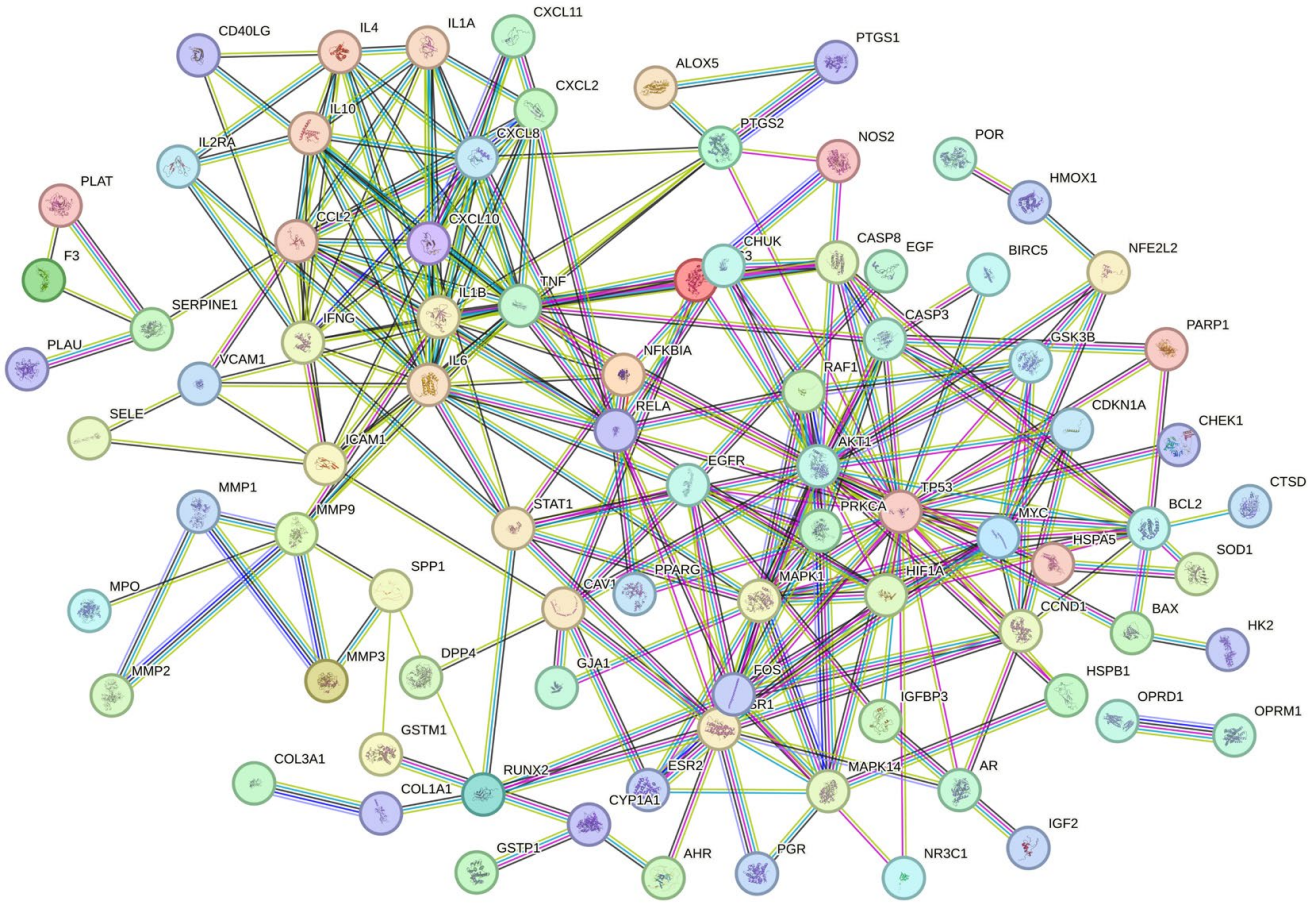
PPI Network Diagram of Common Genes between EMS and OA.

**FIGURE 3:**
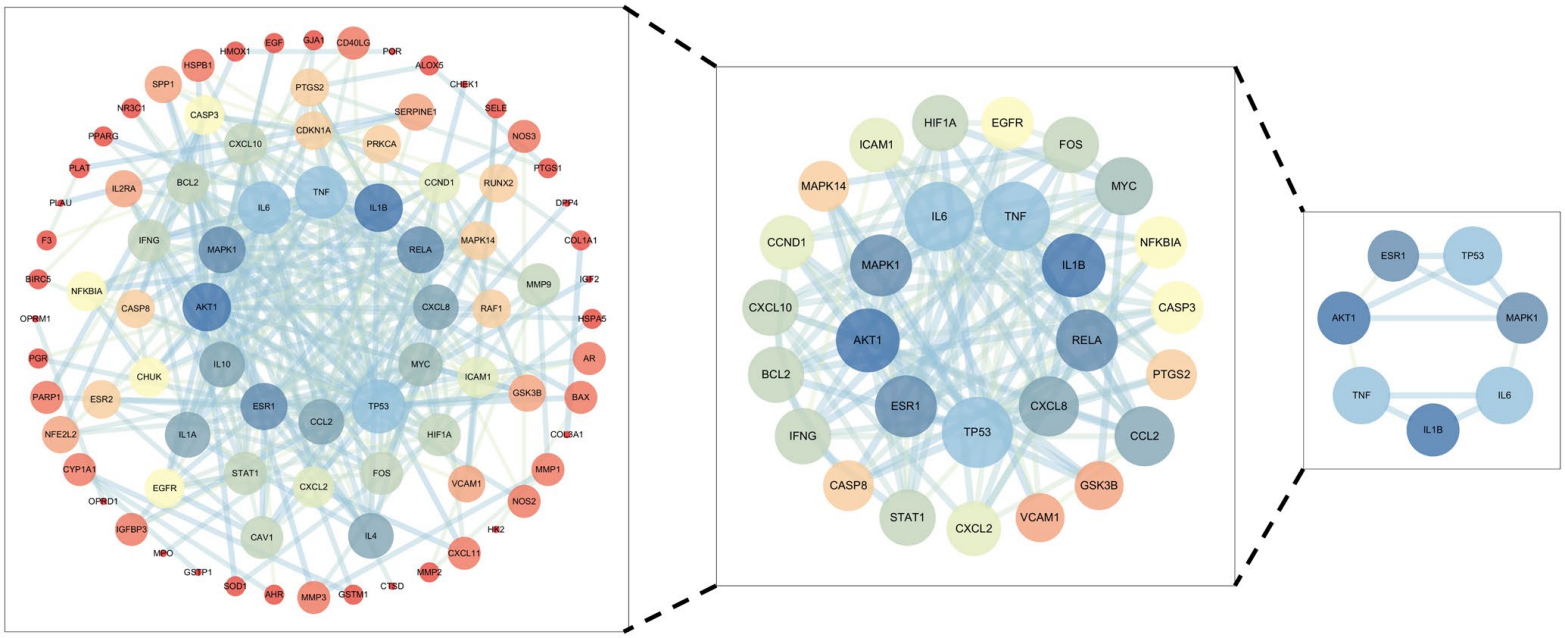
PPI topology analysis.

Potential targets of EMS for treating OA were input into the STRING 12.0 database, selecting “ *Homo sapiens* ” as the species and setting the confidence level to 0.900. After removing disconnected nodes, a PPI network was constructed, consisting of 85 nodes and 260 edges (Figure 2). The cytoNCA plugin was used to analyze network properties, yielding median values of Betweenness, Closeness, Degree, Eigenvector, LAC, and Network scores as 22.640, 0.205, 4, 0.036, 1.5, and 2, respectively. Using these medians as thresholds, 29 candidate targets were identified. Further filtering with the criteria Betweenness: 228.372, Closeness: 0.225, Degree: 11, Eigenvector: 0.135, LAC: 4.333, and Network: 6.451 resulted in seven core targets (Figure 3). The target proteins were prepared for docking with the primary active ingredients that were previously analyzed.

### 3.5 GO Function and KEGG Pathway Enrichment Analysis

**FIGURE 4:**
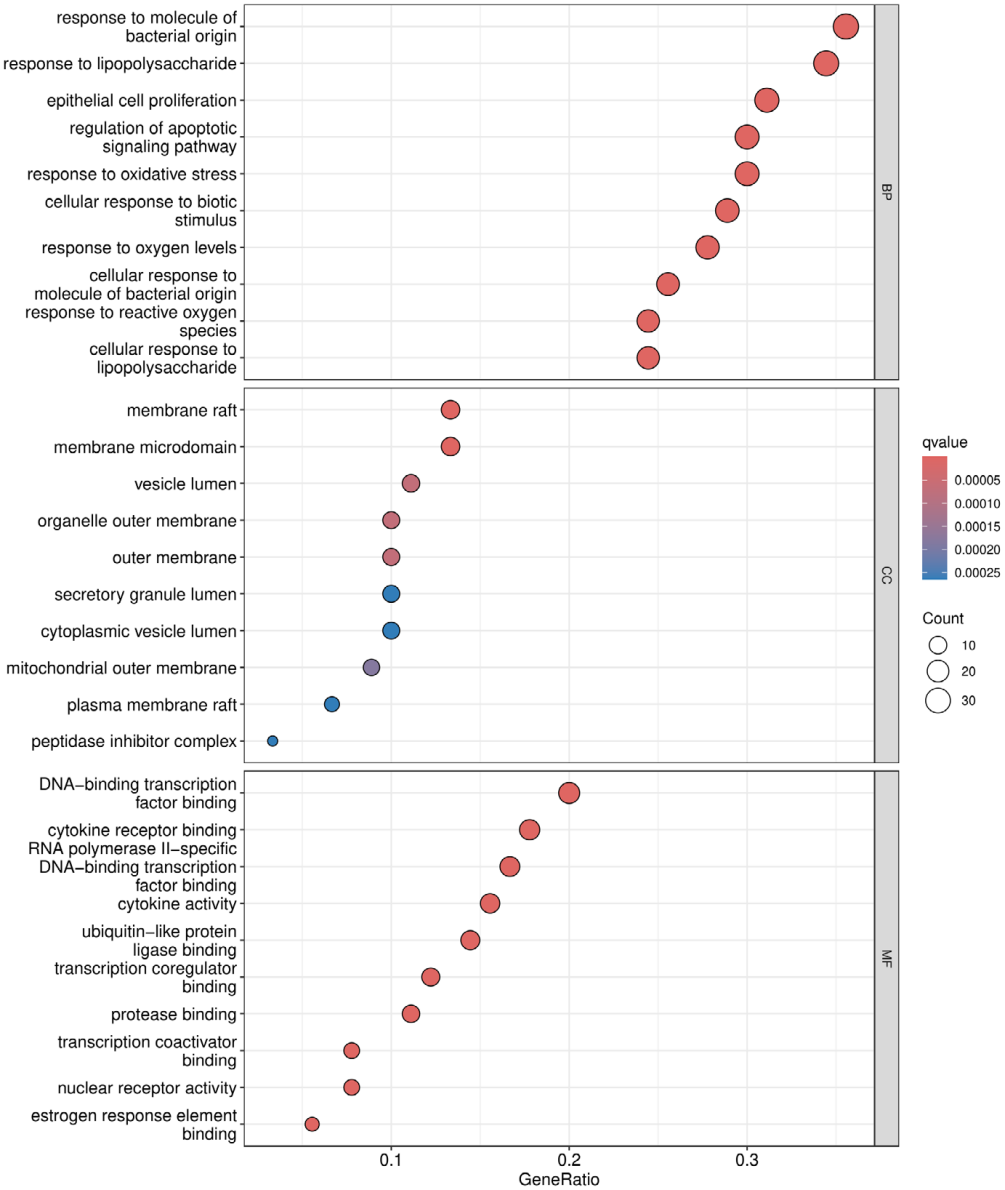
GO enrichment analysis.

After converting the 90 common genes shared between EMS and OA into ID numbers, GO functional enrichment analysis and KEGG pathway enrichment analysis were conducted using R software packages. The GO enrichment analysis identified 2273 entries, with key processes related to response to bacterial molecules, lipopolysaccharide, epithelial cell proliferation, and regulation of apoptosis and oxidative stress. For CC, 40 entries were identified, primarily involving membrane rafts, membrane microdomains, vesicle lumen, organelle outer membrane, and outer membrane. The MF analysis identified 137 entries, focusing mainly on DNA‐binding transcription factor binding, cytokine receptor binding, RNA polymerase II‐specific DNA‐binding transcription factor binding, cytokine activity, and ubiquitin‐like protein ligase binding. A bubble chart (Figure 4) was used to visualize the top 10 terms. The dots with a redder color indicate a lower q‐value and a higher enrichment of the corresponding GO term.

**FIGURE 5:**
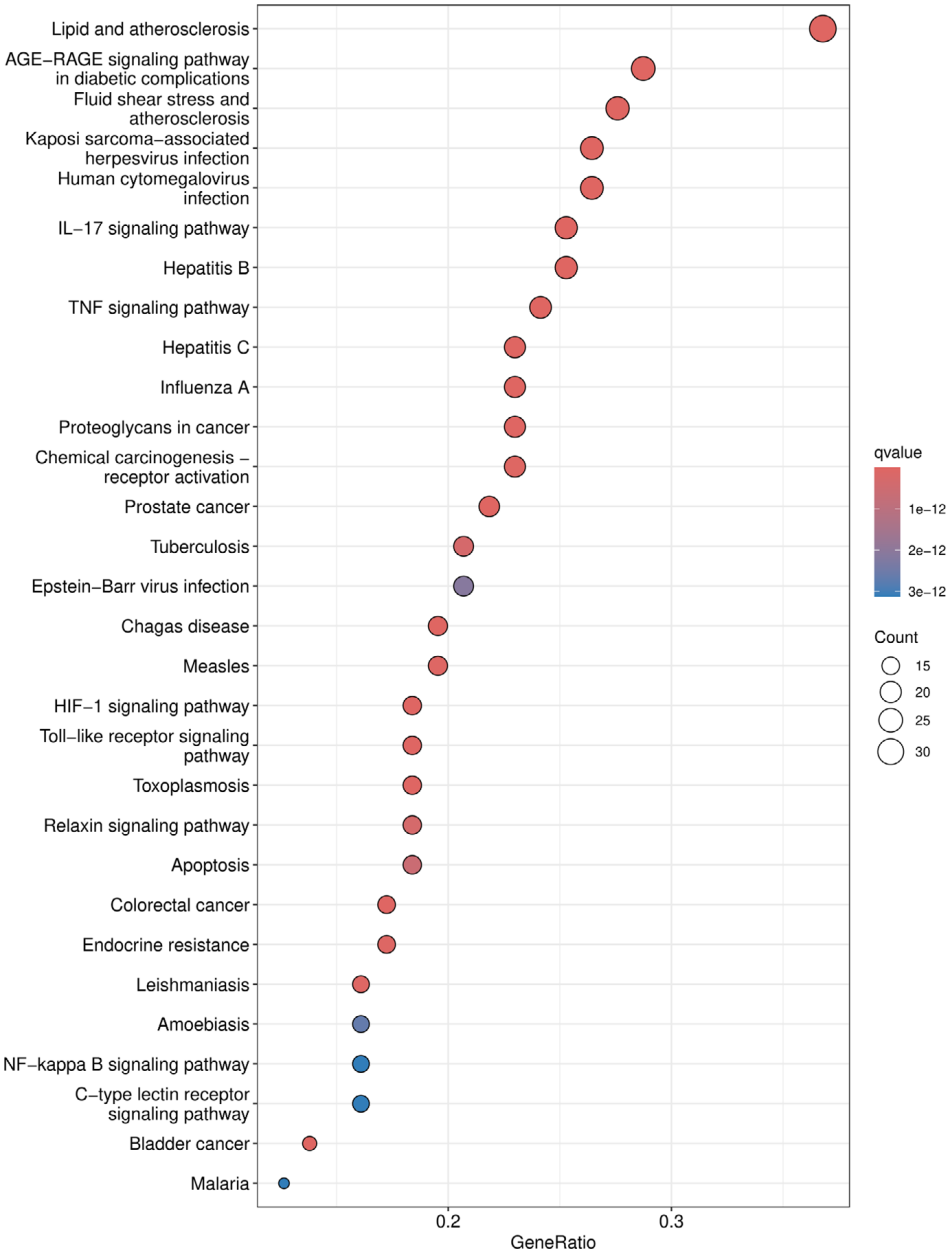
KEGG pathway bubble map.

The KEGG pathway enrichment analysis revealed 167 pathways in total. Among these, the top 30 pathways with the highest degree of enrichment were selected for further investigation. These included pathways associated with lipid metabolism, atherosclerosis, the IL‐17 signaling pathway, and the TNF signaling pathway (Figure 5).

### 3.6 Docking Study

Molecular docking analysis assessed the binding affinity of bioactive compounds to key targets, simulating interactions between receptor proteins and ligands. Negative binding‐free energies denote spontaneous ligand–receptor association. Affinities below −5.0 kcal mol^−1^ are conventionally classified as high; progressively more negative values indicate correspondingly stronger binding (Wang et al. 2025). Notably, the docking results revealed that compounds such as quercetin, rutaecarpine, and beta‐sitosterol exhibited strong binding affinities to core OA‐related targets including TNF, IL‐6, and AKT1 (Table 3). For the top‐scoring complex, Rutaecarpine–TNF (Vina score = −10.0 kcal/mol), the ligand was snugly accommodated within the binding cavity, forming critical hydrogen bonds and extensive hydrophobic contacts. Similarly, Quercetin demonstrated robust binding to TNF (Vina score = −9.5 kcal/mol), primarily stabilized by multiple hydrogen bonds between its phenolic hydroxyl groups and polar residues in the active site, as well as π‐π stacking with aromatic side chains. The strong binding of Beta‐sitosterol to TP53 (Vina score = −8.5 kcal/mol) was predominantly driven by hydrophobic and van der Waals interactions, effectively fitting into the protein's hydrophobic pocket. These interactions are consistent with the high binding energies observed, suggesting a stable and specific binding mode that may underlie the anti‐inflammatory and chondroprotective effects of EMS.

**TABLE 3:** Docking results of five bioactive ingredients from EMS for OA targets.

Ingredients	Vina score						
	TP53	IL6	TNF	IL1B	AKT1	ESR1	MAPK1
Quercetin	−8.2	−6.5	−9.5	−6.9	−8.8	−8.3	−7.2
Wogonin	−7.1	−6.6	−8.4	−6.5	−6.0	−5.9	−7.6
Beta‐sitosterol	−8.5	−6.6	−9.5	−7.5	−7.5	−6.6	−6.7
Dehydrotanshinone II A	−8.2	−7.5	−9.3	−7.5	−7.1	−8.2	−7.5
Rutaecarpine	−8.3	−7.2	−10.0	−7.3	−7.1	−8.6	−7.8

**FIGURE 6:**
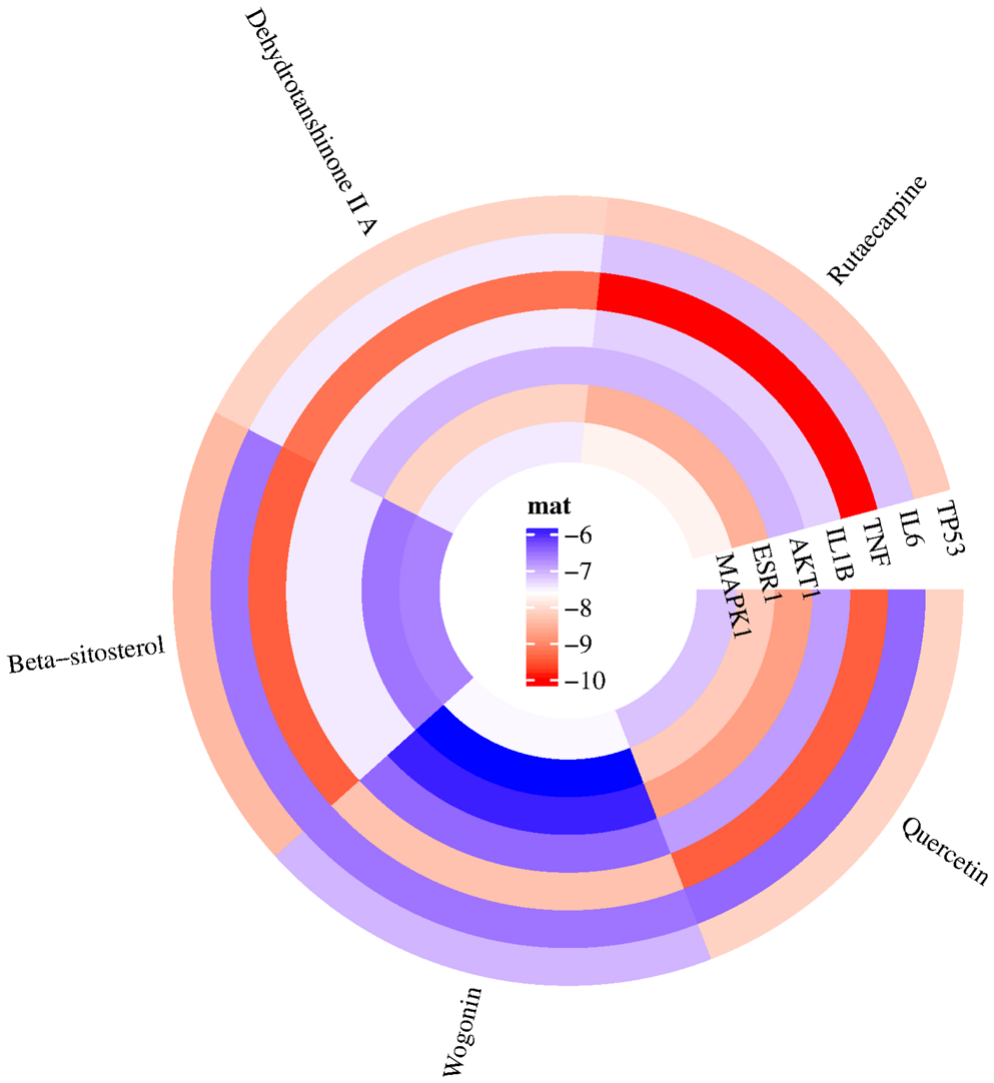
Molecular docking heatmap.

**FIGURE 7:**
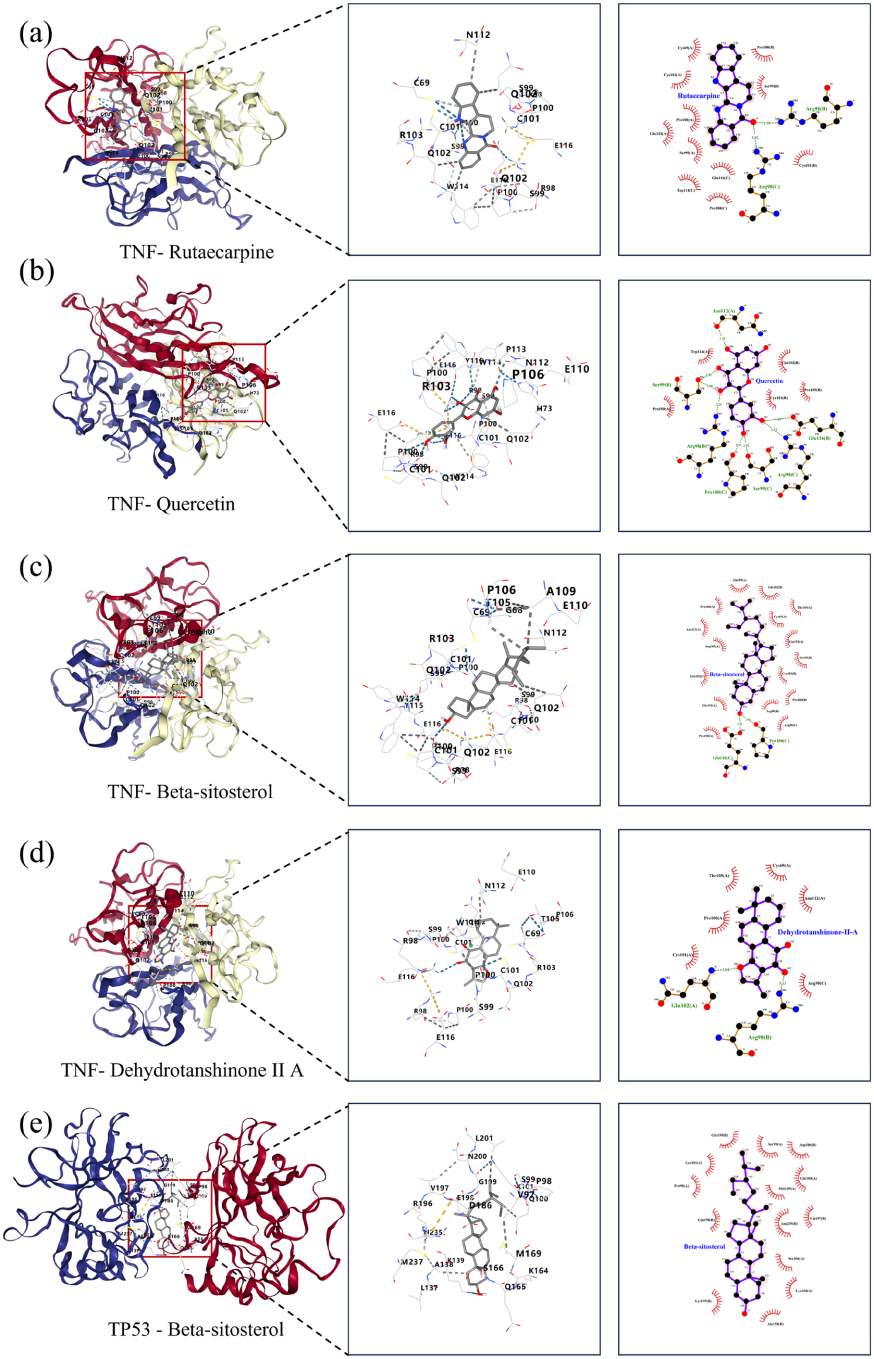
Molecular docking and 2D interaction map of compounds with receptor proteins. (A) TNF ‐ Rutaecarpine; (B) TNF ‐ Quercetin; (C) TNF ‐ Beta ‐ sitosterol; (D) TNF ‐ Dehydrotanshinone II A; (E) TP53 ‐ Beta ‐ sitosterol.

Furthermore, heat maps (Figure 6) were utilized to visually represent the molecular docking outcomes. The heatmap was generated using https://www.bioinformatics.com.cn, an online platform for data analysis and visualization. The docking results with the five highest binding energies are shown in Figure 7 and Table 4.

**TABLE 4:** Docking results for the top five binding energies.

Complexes	Vina score (kJ/mol)	Cavity volume (Å3)	Center (*x*, *y*, *z*)	Docking size (*x*, *y*, *z*)
TNF—Rutaecarpine	−10	1038	−2, −7, 3	21, 21, 21
TNF—Quercetin	−9.5	1038	−2, −7, 3	21, 21, 21
TNF—Beta‐sitosterol	−9.5	1038	−2, −7, 3	25, 25, 25
TNF—Dehydrotanshinone II A	−9.3	1038	−2, −7, 3	20, 20, 20
TP53—Beta‐sitosterol	−8.5	686	117, 84, −31	25, 25, 25

### 3.7 Molecular Dynamics Simulation

**FIGURE 8:**
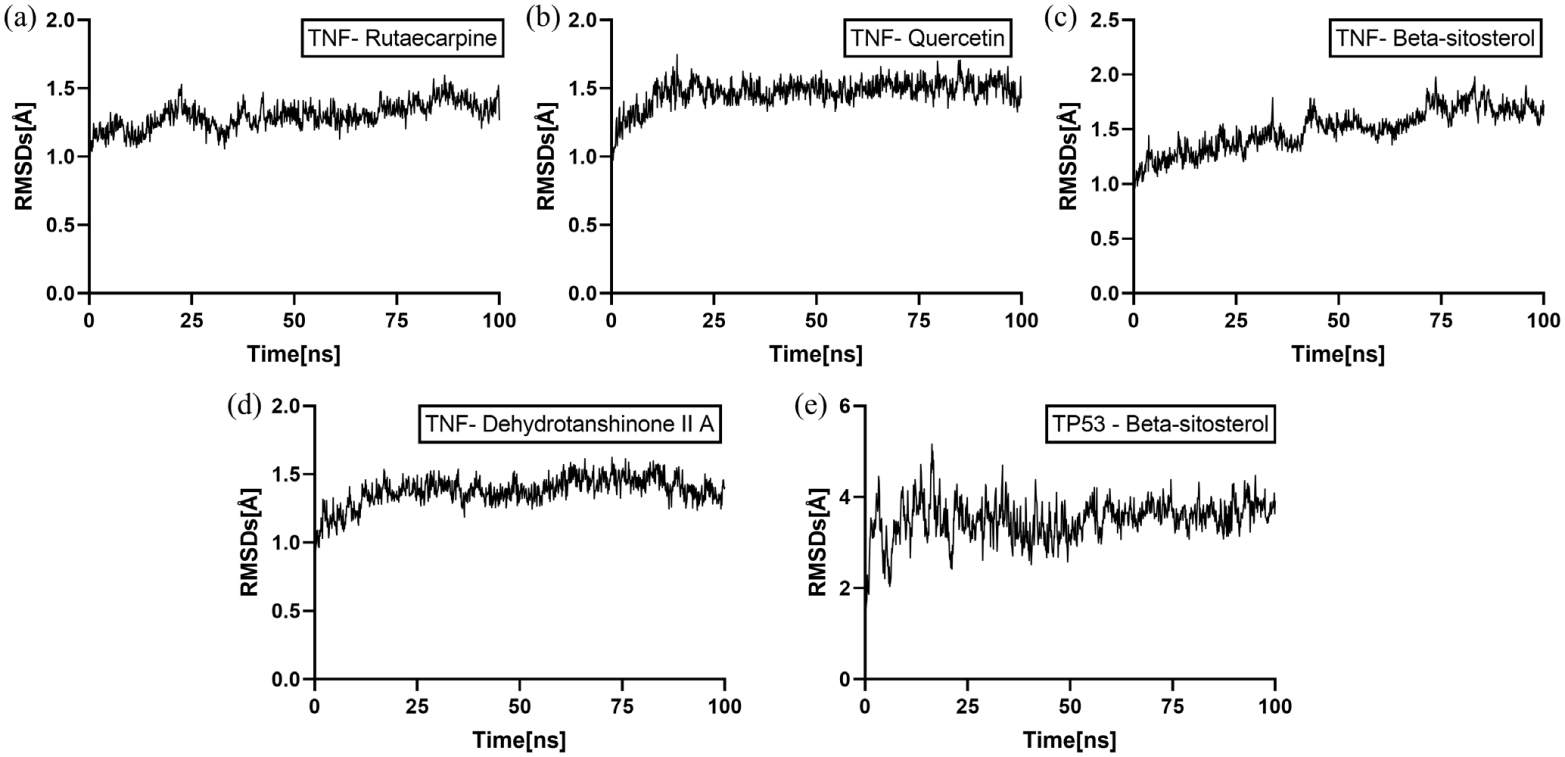
Temporal variation in RMSD values for protein–ligand complexes. (A) TNF ‐ Rutaecarpine; (B) TNF ‐ Quercetin; (C) TNF ‐ Beta ‐ sitosterol; (D) TNF ‐ Dehydrotanshinone II A; (E) TP53 ‐ Beta ‐ sitosterol.

To further validate the binding affinity and stability of the ligand‐target complexes identified from molecular docking with the top five binding energies, molecular dynamics (MD) simulations were performed on these targets in descending order of binding‐free energy. The RMSD serves as an effective metric for assessing the conformational stability of proteins and ligands, reflecting the extent of deviation in atomic coordinates from their initial configurations. Lower RMSD values indicate higher conformational stability (Coutsias and Wester 2019). RMSD was employed to evaluate the stability of the simulated systems. Each RMSD plot includes a black trace representing the fluctuation of the backbone atoms of the receptor‐target protein complex. The MD simulation results (Figure 8) depict the RMSD values for Rutaecarpine, Quercetin, Beta‐sitosterol, and Dehydrotanshinone II A bound to the TNF target protein, as well as Beta‐sitosterol bound to the TP53 target protein, over a 100 ns simulation period. Quercetin complexed with TNF exhibited the highest conformational stability, maintaining an RMSD consistently below 3.0 Å throughout the simulation with less fluctuation compared to the other complexes, indicating a particularly stable interaction structure (Figure 8b). This observation aligns with the molecular docking results showing strong binding energy. In contrast, the Beta‐sitosterol‐TP53 complex displayed the greatest RMSD fluctuation (Figure 8e). These simulation results not only corroborate the molecular docking findings but also provide critical dynamic insights into the stability of the core ligand‐target complexes, thereby offering a more reliable theoretical foundation for understanding the mechanism of EMS.

### 3.8 Pharmacokinetics Study

**FIGURE 9:**
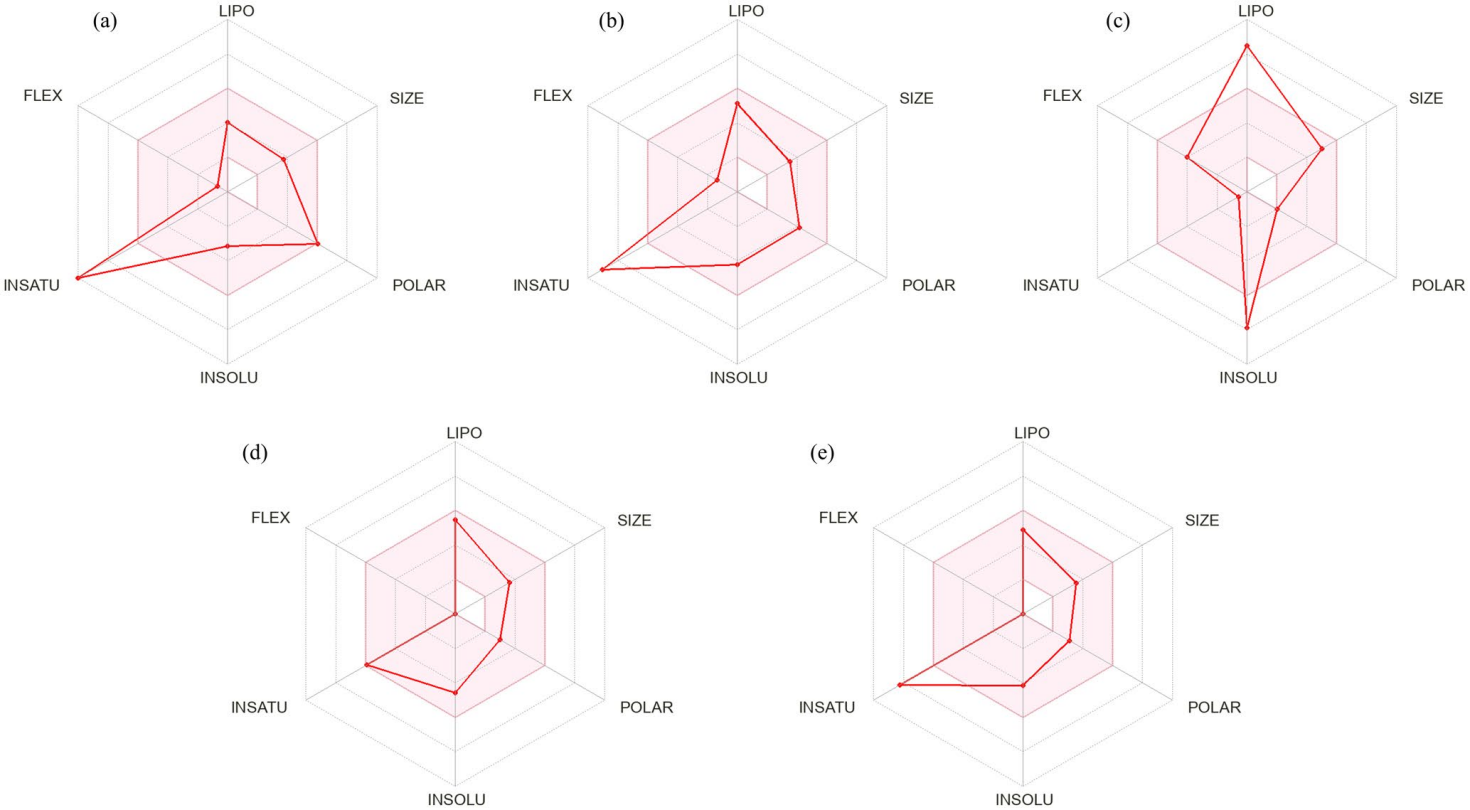
Radar plot of predicted physicochemical properties for the top five compounds (A) Quercetin; (B) Wogonin; (C) Beta‐sitosterol; (D) Dehydrotanshinone II A; (E) Rutaecarpine.

The radar plot in Figure 9 illustrates six physicochemical properties of five compounds revealing significant differences in their lipid solubility (LIPO), molecular size, polarity (POLAR), insolubility (INSOLU), insaturation (INSATU), and flexibility (FLEX). Quercetin exhibited high polarity and insaturation, while beta‐sitosterol had high lipid solubility and insolubility, and wogonin scored high on insaturation. The differences may be related to the chemical structure of the molecules and their functional groups.

Based on SwissADM server, Table 5 summarizes the pharmacokinetic properties of the five studied compounds. Quercetin, wogonin, dehydrotanshinone II A, and rutaecarpine exhibited high gastrointestinal absorption, whereas beta‐sitosterol showed low gastrointestinal absorption. Regarding P‐glycoprotein (P‐gp) substrate status, rutaecarpine was confirmed as a P‐gp substrate, suggesting the possibility of efflux by P‐gp transporters, which could affect its bioavailability and tissue distribution. Furthermore, beta‐sitosterol did not exhibit any inhibitory effects on cytochrome P450 enzymes. In contrast, the other four compounds demonstrated varying inhibitory effects on different cytochrome P450 enzymes. Among them, quercetin and rutaecarpine exhibited similar inhibitory profiles, both inhibiting CYP1A2, CYP2D6, and CYP3A4.

**TABLE 5:** The predicted pharmacokinetics of EMS.

Pharmacokinetics	Quercetin	Wogonin	Beta‐sitosterol	Dehydrotanshinone II A	Rutaecarpine
GI absorption	High	High	Low	High	High
BBB permeant	No	No	No	Yes	Yes
P‐gp substrate	No	No	No	No	Yes
CYP1A2 inhibitor	Yes	Yes	No	Yes	Yes
CYP2C19 inhibitor	No	No	No	Yes	No
CYP2C9 inhibitor	No	Yes	No	Yes	No
CYP2D6 inhibitor	Yes	Yes	No	No	Yes
CYP3A4 inhibitor	Yes	Yes	No	Yes	Yes
Log Kp (skin permeation)	−7.05 cm/s	−5.56 cm/s	−2.20 cm/s	−5.21 cm/s	−5.90 cm/s

*Note:* CYP1A2, CYP2C19, CYP2C9, CYP2D6 and CYP3A4 are the five forms of cytochromes P450 (CYP).

Abbreviations: BBB, blood brain barrier; GI, gastrointestinal; P‐gP, P‐glyco‐protein transporter.

## 4 Discussion

The pathogenesis of OA is complex and varied and has not yet been fully defined. However, the disease seriously affects the quality of life of patients. Non‐surgical therapeutic measures, such as non‐steroidal anti‐inflammatory drugs (NSAIDs), intra‐articular corticosteroid injections and hyaluronic acid, cannot stop the progression of OA, although they can alleviate clinical symptoms to a certain extent. With the popularization and rapid development of TCM, significant progress has been made in the treatment of OA with TCM and it is widely welcomed by patients.

Key bioactive compounds from EMS—including quercetin, wogonin, beta‐sitosterol, dehydrotanshinone II A, and rutaecarpine—converge upon a central hub of core protein targets. Quercetin, a natural flavonoid widely found in vegetables and fruits, demonstrates significant potential in modulating exercise‐induced oxidative stress (Özdemir and Demir 2025). Zor et al. (Zor et al. 2025) identify quercetin as a key phenolic component within the bioactive profile of *Elaeagnus angustifolia* plant extracts. The study highlights its contribution to the extract's overall enzyme inhibitory properties, showing notable activity against critical enzymes such as acetylcholinesterase (AChE) and tyrosinase. Li et al. (Li et al. 2021) found that quercetin inhibited IL‐1β‐induced inflammatory response and cartilage degradation, reduced inflammation and apoptosis, and down‐regulated the expression levels of IRAK1, NLRP3, and cysteinyl asparagin‐3 by inhibiting the IRAK1/NLRP3 signaling pathway. Consistent with these findings, our molecular docking results demonstrated that quercetin exhibits strong binding affinity to key inflammatory targets such as TNF and AKT1, providing a structural basis for its purported mechanism. A study by Khan et al. further revealed the significant role of wogonin in the enhancement of chondrocyte viability in human OA (Khan et al. 2017). They found that under the stimulation of IL‐1β, wogonin was able to effectively regulate key signaling pathways such as NF‐κB, ERK1/2, JNK1/2, and p38 MAPKs, which in turn affected the expression of genes related to the cartilage formation phenotype. Lou et al.'s research demonstrated that beta‐sitosterol, an active component of oat‐derived (OD) supplements, can reduce inflammation and cartilage degeneration in OA by suppressing chondrocyte apoptosis and the activation of the MAPK signaling pathway (Lou et al. 2023). However, clinical trials on the use of beta‐sitosterol in the treatment of OA have not yet been initiated, and further clinical trials will help to validate its safety and efficacy in patients with OA and provide a stronger evidence‐based rationale for its use. Dehydrotanshinone II A has garnered global attention for its potential as a standalone treatment or in combination with other medications for dual/combined therapy (Ansari et al. 2021). Zhou et al. (2021) have demonstrated that dehydrotanshinone II A mitigates inflammation in OA by inhibiting the miR‐155/FOXO3 signaling pathway. Rutaecarpine, a bioactive alkaloid derived from Evodia rutaecarpa, has recently been identified as an agent that alleviates OA symptoms. This compound exerts its therapeutic effects by suppressing the PI3K/AKT/NF‐κB and MAPK signaling pathways, which are mediated through the integrin αVβ3 receptor (Wan et al. 2023).

Our molecular docking results strongly support the high‐affinity binding of these compounds to targets like TNF, IL6, IL1B, AKT1, and TP53. Notably, TNF emerged as a common high‐affinity target for multiple components, suggesting its role as a pivotal regulatory node through which EMS exerts a significant portion of its anti‐inflammatory effect. TP53, a tumor suppressor gene, plays a crucial role in cellular apoptosis and DNA repair mechanisms (George et al. 2021). TNF‐α, IL‐1β, and IL‐6 are key inflammatory mediators involved in the inflammatory response and influence the progression of OA. Studies have shown that TNF‐α can bind to TNFR1 and TNFR2 receptors to form a complex, which in turn activates the NF‐κB and MAPK signaling pathways, thus promoting the production of inflammatory factors (Zhang et al. 2021). The mechanism of action of IL‐1 in cartilage degeneration in OA mainly includes: promoting the apoptotic process of chondrocytes, inhibiting the synthesis of cartilage matrix, inducing the up‐regulation of the expression of matrix metalloproteinases (MMPs), and participating in the release of inflammatory mediators (Fei et al. 2019). IL‐6 is regarded as a cytokine that promotes the inflammatory response, and its interaction with IL‐1β and TNF‐α exacerbates the inflammatory process (Wojdasiewicz et al. 2014). AKT1, a pivotal downstream effector kinase in the PI3K/AKT signaling pathway, is implicated in various pathological processes, including cell proliferation, apoptosis, and inflammatory responses (Chen et al. 2021). The upregulation of AKT1 has been shown to enhance the expression of IL‐1β and TNF‐α, thereby accelerating the progression of inflammation. ESR1, a receptor for estrogen, modulates gene expression and function in conjunction with estrogen (Huang et al. 2021). Polymorphisms in ESR1 are utilized to investigate the genetic influences on OA.

The subsequent signaling tier involves the modulation of critical OA‐related pathways by these core targets. KEGG enrichment analysis points to a coordinated suppression of the TNF signaling pathway and the IL‐17 signaling pathway, which are master regulators of inflammation. This likely leads to the downregulation of downstream inflammatory mediators and enzymes such as COX‐2 (PTGS2). Simultaneously, components like rutaecarpine and beta‐sitosterol appear to dampen the PI3K‐AKT signaling pathway and the MAPK signaling pathway (e.g., via AKT1 and MAPK1). The significance of this dual inhibition lies in the well‐established crosstalk between these pathways and NF‐κB signaling, creating a powerful, synergistic circuit that potently suppresses inflammation and chondrocyte apoptosis.

This coordinated molecular and signaling interference culminates in protective cellular effects. The collective inhibition of the TNF, IL‐17, PI3K‐AKT, and MAPK pathways directly translates into a marked reduction in the inflammatory response and the expression of matrix‐degrading enzymes (MMPs). Furthermore, the modulation of these pathways, along with targets like TP53, contributes to diminishing chondrocyte apoptosis and alleviating oxidative stress. The net result of these concerted actions is the preservation of the extracellular matrix and the protection of cartilage integrity. Researchers have found that lipid deposition in the joints is observed in the early stages of OA, and several studies have shown that the progression of OA is strongly associated with disorders of lipid metabolism (Gkretsi et al. 2011; Kruisbergen et al. 2022). Iijima et al. (2022) showed a close relationship between the AGE‐RAGE signaling pathway and age‐related OA in diabetic complications. IL‐17 activates the NF‐κB signaling pathway, which is involved in the pathological process of OA and is a key factor in OA, suggesting that its signaling pathway is a potential target for OA treatment (Lepetsos et al. 2019). The TNF signaling pathway is associated with the pathogenesis of OA, and the aberrant expression of TNFα promotes the disease progression and synergistically promotes the ECM degradation and inflammation with IL‐1β (Luo et al. 2023).

The SwissADME Server is a user‐friendly web tool for physicochemical property, pharmacokinetic, drug similarity and medicinal chemistry analyses that allows rapid prediction of key parameters for a range of molecules (Daina et al. 2017). We applied the server to analyze the pharmacokinetic properties of the five studied compounds screened. Among them, the four active ingredients, quercetin, wogonin, dehydrotanshinone II A, and rutaecarpine, all showed a high degree of gastrointestinal absorption, which provided some theoretical basis for the administration of EMS. As P‐gp is an efflux transporter highly expressed in the intestinal epithelium, its activity can limit the oral bioavailability and tissue distribution of its substrates (Zhang et al. 2025). This suggests that the systemic exposure to rutaecarpine after oral administration of EMS might be constrained, which could necessitate further formulation strategies to overcome this limitation for optimizing its therapeutic efficacy. Equally important are the inhibitory effects of the key components on various cytochrome P450 (CYP) enzymes. Quercetin and rutaecarpine were predicted to inhibit CYP1A2, CYP2D6, and CYP3A4, while dehydrotanshinone II A and wogonin also showed inhibition profiles against several CYPs, particularly CYP3A4. Since CYP3A4 is involved in the metabolism of a vast number of clinically used drugs, concurrent administration of EMS with drugs that are substrates of these enzymes (e.g., certain calcium channel blockers, statins, or immunosuppressants) could potentially lead to herb–drug interactions by reducing the metabolic clearance of the coadministered drugs, thereby increasing their plasma levels and the risk of adverse effects (Matsson et al. 2025). These findings highlight the necessity for future investigations into the safety profile of EMS, especially concerning its combination with conventional pharmaceuticals, to ensure its safe and effective use in a clinical setting.

In this study, we investigated the therapeutic mechanism of EMS on OA using a network pharmacology approach, revealing that the therapeutic effects on OA are realized through multiple components, pathways, and targets. Although EMS has demonstrated significant therapeutic value, attention must be paid to its pharmacological limitations, such as the poor solubility of beta‐sitosterol. Furthermore, research has found that quercetin is unstable in heat, light, and air, which may affect its use in formulations (Yin et al. 2018; Samadi et al. 2022). Fortunately, the application of small molecule drug modification is progressing. Through the application of targeted carriers and nanotechnology, researchers have developed a variety of methods to improve the physical properties of small molecules, thereby increasing drug utilization and efficacy. For example, the solubility, stability, and targeting of small molecule drugs can be enhanced through carrier systems such as nanoparticles, liposomes, and polymeric nanodrugs (Cong et al. 2022; Xiao et al. 2021; Tang et al. 2023). In addition, through the structural modification and optimization of active ingredients in TCM, combined with modern science and technology, the research and development of new drugs can be accelerated to provide more solid theoretical support and scientific basis for the treatment of OA by TCM.

Although this study theoretically explored the potential therapeutic mechanism of EMS, there are limitations. Future investigations should include more detailed animal experiments and Phase I clinical trials to comprehensively evaluate the safety, efficacy, and pharmacokinetic profile of EMS in OA patients. These studies will verify its actual efficacy on OA and provide more solid theoretical support and scientific evidence for the treatment of OA with TCM.

## 5 Conclusion

This study integrated network pharmacology, molecular docking and molecular dynamics simulations to systematically elucidate the potential mechanism of EMS in treating osteoarthritis, identifying key active compounds such as quercetin, wogonin, and rutaecarpine, and revealing their synergistic effects on core targets including TNF, IL‐6, and AKT1. Molecular docking demonstrated strong binding affinities between these compounds and the core targets, particularly with TNF, suggesting it may serve as a central target for the anti‐OA effects of EMS. However, these computational predictions require experimental validation. The next steps should include conducting in vivo studies using animal models of OA to verify therapeutic efficacy and observe downstream effects on predicted targets and pathways, ultimately progressing to well‐designed clinical trials to translate these findings into evidence‐based OA treatments.

In summary, this study deciphers the complex “multi‐component, multi‐target, multi‐pathway” mechanism of EMS against OA, providing a scientific rationale and specific targets for future research and drug development.

## Author Contributions

**Zhenyu Song:** conceptualization (lead), data curation (lead), formal analysis (lead), funding acquisition (lead), investigation (lead), methodology (lead), project administration (supporting), resources (lead), software (lead), supervision (supporting), validation (lead), visualization (lead), writing – original draft (lead), writing – review and editing (equal). **Jincheng Huang:** conceptualization (equal), data curation (supporting), formal analysis (supporting), investigation (supporting), methodology (supporting), project administration (supporting), supervision (lead), validation (lead), visualization (supporting), writing – review and editing (supporting). **He Zhu:** methodology (supporting). **Xu Li:** formal analysis (supporting). **Qianqian Cao:** data curation (supporting). **Liyang Yang:** conceptualization (equal), software (equal). **Meng Zhang:** formal analysis (equal), project administration (equal), validation (equal). **Hongkai Wang:** investigation (supporting), methodology (supporting). **Haoyue Sun:** investigation (supporting), methodology (supporting), project administration (supporting), writing – review and editing (supporting).

## Funding

This work was supported by Advanced Scientific Research Foundation for the Returned Overseas Chinese Scholars in Henan Province (2024HNSLXRY08); Key Scientific and Technological Projects in Henan Province (LHGJ20210024); National Natural Science Foundation of China (No. 82002840); Guangxi Medical and Health Key Cultivation Discipline Construction Project [Guiwei Kejiao Fa 2022 No. 4].

## Ethics Statement

The authors have nothing to report.

## Conflicts of Interest

The authors declare no conflicts of interest.

## Data Availability

All data supporting the article are provided in this article.
